# Pushing nuclear magnetic resonance sensitivity limits with microfluidics and photo-chemically induced dynamic nuclear polarization

**DOI:** 10.1038/s41467-017-02575-0

**Published:** 2018-01-09

**Authors:** Miguel Mompeán, Rosa M. Sánchez-Donoso, Antonio de la Hoz, Vittorio Saggiomo, Aldrik H. Velders, M. Victoria Gomez

**Affiliations:** 1 Instituto Regional de Investigación Científica Aplicada (UCLM), Avda Camilo José Cela s/n, 13071 Ciudad Real, Spain; 2 Laboratory of BioNanoTechnology, Wageningen University, PO Box 8038, 6700 EK Wageningen, The Netherlands; 3 MAGNEtic resonance research FacilitY–MAGNEFY, Wageningen University & Research, PO Box 8038, 6700 EK Wageningen, The Netherlands

## Abstract

Among the methods to enhance the sensitivity of nuclear magnetic resonance (NMR) spectroscopy, small-diameter NMR coils (microcoils) are promising tools to tackle the study of mass-limited samples. Alternatively, hyperpolarization schemes based on dynamic nuclear polarization techniques provide strong signal enhancements of the NMR target samples. Here we present a method to effortlessly perform photo-chemically induced dynamic nuclear polarization in microcoil setups to boost NMR signal detection down to sub-picomole detection limits in a 9.4T system (400 MHz ^1^H Larmor frequency). This setup is unaffected by current major drawbacks such as the use of high-power light sources to attempt uniform irradiation of the sample, and accumulation of degraded photosensitizer in the detection region. The latter is overcome with flow conditions, which in turn open avenues for complex applications requiring rapid and efficient mixing that are not easily achievable on an NMR tube without resorting to complex hardware.

## Abstract (web summary)

Nuclear magnetic resonance (NMR) spectroscopy is a powerful technique with an inherently low sensitivity. Here, the authors present a combination of microcoils with photo-chemically induced dynamic nuclear polarization to boost NMR sensitivity down to sub-picomole detection limits.

## Introduction

Despite its low sensitivity, nuclear magnetic resonance (NMR) spectroscopy is undoubtedly one of the most powerful analytical techniques to obtain unique information on (macro)molecular composition, structure and dynamics^1^. Strategies to improve sensitivity comprise use of extreme magnetic fields, cryoprobe technologies^2^ and dynamic nuclear polarization (DNP) techniques^3^, which however all go hand in hand with enormous costs and requirements for high tech equipment. Alternatively, microcoils have proven to be a most cost-effective approach to tackle mass sensitivity on the basis that sensitive signal detection depends on the sample/coil volume ratio; however, concentration sensitivity still remains an issue. Here we show that the combination of microcoils with photo-chemically induced dynamic nuclear polarization (photo-CIDNP) provides a breakthrough in both mass and concentration sensitivity enhancements of small-volume NMR and a concomitant renaissance of the photo-CIDNP technique, that to date has been limited to specialized labs. This setup provides low-cost components and excellent performance at the moderate magnetic field strengths of 9.4T, rivalling performance limits of state-of-the-art cryoprobes at much higher field strengths.

Miniaturized NMR coils are promising tools to both enhance the amplitude of the NMR signal^4–13^ and to enable the study of mass-limited samples in a number of applications. In particular, glass chips with integrated planar spiral microcoils^11, 12^ have proven most versatile, and have enabled us the study of supramolecular interactions^14^ and online monitoring of isothermal and non-isothermal chemical reactions^15, 16^. Still, glass-based microfluidic NMR chips require clean-room technology and hence are relatively time-consuming and expensive in manufacturing, hampering the advancement and more wide-spread use of small-volume NMR technologies. In this regard, 3D-printed and polymer-based microfluidic NMR devices are rapidly and more readily made with low-cost materials and without need for clean-room technology^17–19^. We envisioned that flexible and transparent polymeric material, like our PDMS device^19^, was likely to enable uniform irradiation with low-power light sources to allow the development of new setups for photo-CIDNP on small-volume systems to tackle the concentration sensitivity limitations of such devices.

The CIDNP effect occurs as enhanced NMR signals in a target molecule that forms a radical pair (RP) with a photoexcited photosensitizer (commonly a flavin). Not all the nuclei of a molecule engaged in such an RP will experience the hyperpolarization phenomenon, but only those experiencing notable hyperfine interactions with the unpaired electron^20, 21^. This is important because, for historical reasons, the acronym DNP is contained within CIDNP, which might yield confusion. We recall that in solution DNP the enhancements can be experienced by all nuclei in a given molecule, as the dominant mechanism is the Overhauser transfer of the large electron spin polarization onto the nuclear spins; therefore, limiting the maximum theoretical enhancements to the electron/nucleus gyromagnetic ratio (~ 660 for proton)^22^. In CIDNP, the RP mechanism operates in a distinct way, where the enhancements are usually proportional to the hyperfine coupling constant^23–25^ and sometimes to its second or even third power in aromatic compounds^24^. Two major traditional challenges of photo-CIDNP are lack of uniform irradiation of the whole sample volume and photodegradation of the sensitizer, e.g. flavin. Previous attempts to achieve a more homogeneous irradiation of the hundreds of microlitres contained within a conventional NMR tube dealt with more or less complex modifications of the optical fibre^26–28^. Notably, flavin photodegradation is of high relevance because it limits the number of spectra that can be recorded on a sample^29^. Using H_2_O_2_ to reoxidize the flavin comes at the risk of protein degradation^30^ or oxidation^31^. Therefore, alternative approaches have been devised to minimize the impact of flavin degradation by enhancing the signal in shorter experiments, using complex pulse sequences to store and accumulate the polarizations^32^, or their generation on heteronuclei (^13^C or ^15^N) to be later detected on ^1^H^33, 34^. More recently, the simultaneous use of three enzymes within the solution containing the target sample to reoxidize the flavin has been proposed^35^. The use of LED-based illumination was recently devised to irradiate samples at lower power for conventional 5-mm NMR tubes^36–38^. Nevertheless, the persistence of the bottleneck concerns, and the increasing complexity associated with each of the above improvements devised to surpass them, prompted us for a need of revisiting the method.

**Fig. 1:**
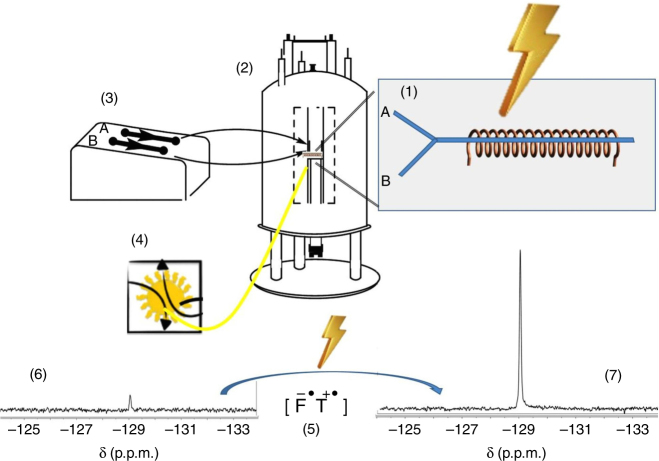
System setup. A solenoidal microcoil **(1)** is embedded within a polydimethylsiloxane (PDMS) matrix and encompassing the outlet part of a double-inlet (‘A’ and ‘B’) Y-shaped channel; the PDMS block fitting in a 3D-printed polylactic acid (PLA) holder. The holder holds a bracket with capacitors connected to the RF circuit and everything is placed inside the magnet **(2)**. Samples can be injected via the two inlets by means of syringe pumps **(3)**. The PDMS matrix is transparent to the visible region of the electromagnetic spectrum allowing efficient irradiation using an optical fibre that guides the light beam from a laser diode positioned outside of the magnet **(4)**. When the target sample (denoted ‘T’) is irradiated in the presence of a photosensitizer (e.g., a flavin, ‘F’), a radical pair **(5)** is formed by a proton or electron abstraction initiated by the photoexcited flavin, and the photo-chemically induced dynamic nuclear polarization (photo-CIDNP) effect results in a dramatic enhancement of the target sample’s NMR signal **(6 and 7)**

In the present manuscript we expand the field of applications of microcoils, showing their suitability to facilitate the performance of photo-CIDNP experiments with a number of advantages that we expect will rekindle the realm of these experiments, all this by virtue of the small active volumes afforded by these devices. The first clear advantage is that high-power light sources are no longer a prerequisite, because our setup requires irradiation of just one microlitre of sample. Moreover, the light source/fibre is implemented in a non-invasive manner, not disturbing the sample detection volume. Finally, the setup allows for straightforward continuous flow photo-CIDNP experiments that, importantly, eliminates the necessity of light pulses, and even allows continuous irradiation. All this dramatically simplifies the experimental setup, with impressive mass and concentration sensitivity, saves measuring times because of elimination of dead/waiting times, and most importantly, resolves the problem of photodegradation products in the detection volume that results in signal loss. The experiments presented here are performed on a 9.4T magnet (400 MHz proton Larmor frequency), yet the home-built setup (Fig. 1) rivals cryoprobe sensitivity at higher fields.

## Results

### Initial remarks

Below, our results are organized as follows: first we describe how the setup as shown in Fig. 1 is simply made and assembled without clean-room technology or specialized equipment. Next, the performance of photo-CIDNP is evaluated using nucleotide and amino acid samples, followed by on-flow experiments monitoring supramolecular interactions as well as folding of polypeptides. Finally, sub-picomole limit-of-detection is presented.

### System setup to perform photo-CIDNP experiments

The setup as shown in Fig. 1 to perform photo-CIDNP experiments was manufactured building on three main components, a light source placed outside the magnet, a microfluidic NMR chip, and an optical fibre to guide the light towards the active volume of the microcoil. To address uniform irradiation of the sample volume, both a current and a temperature controller are used to regulate the amount of output light from a blue laser diode (*λ* = 455 nm) operating in the mW range. The radiofrequency microcoils are incorporated within a polydimethylsiloxane (PDMS) crosslinked polymer defining a microfluidic device or NMR chip that is in turn embedded on a 3D-printed polylactic acid (PLA) holder. An important advantage of PDMS is that it is transparent in the visible light part of the electromagnetic spectrum^39^, which facilitates an efficient light penetration. Accordingly, an optical fibre of 1 mm of internal diameter is inserted within the NMR chip holder, placed 2 mm above the solenoidal coil, to guide the light beam from the laser diode to the detection region of the microcoil containing the sample. This setup decreases heating effects of high-power irradiation and eliminates optical density limitations. High-power illumination (Watts, W) is a requirement in conventional photo-CIDNP experiments to deliver as much light as possible into the hundreds of microlitres of sample inside the NMR tubes. We argue that it is possible to resort to more affordable, low-power lasers operating in the milliWatt (mW) range to perform photo-CIDNP experiments using PDMS-embedded microcoils to facilitate light penetration on the sample. To prove this concept, we used solenoidal microcoils with an active volume of just 1 μL within the framework of the setup presented.

**Fig. 2:**
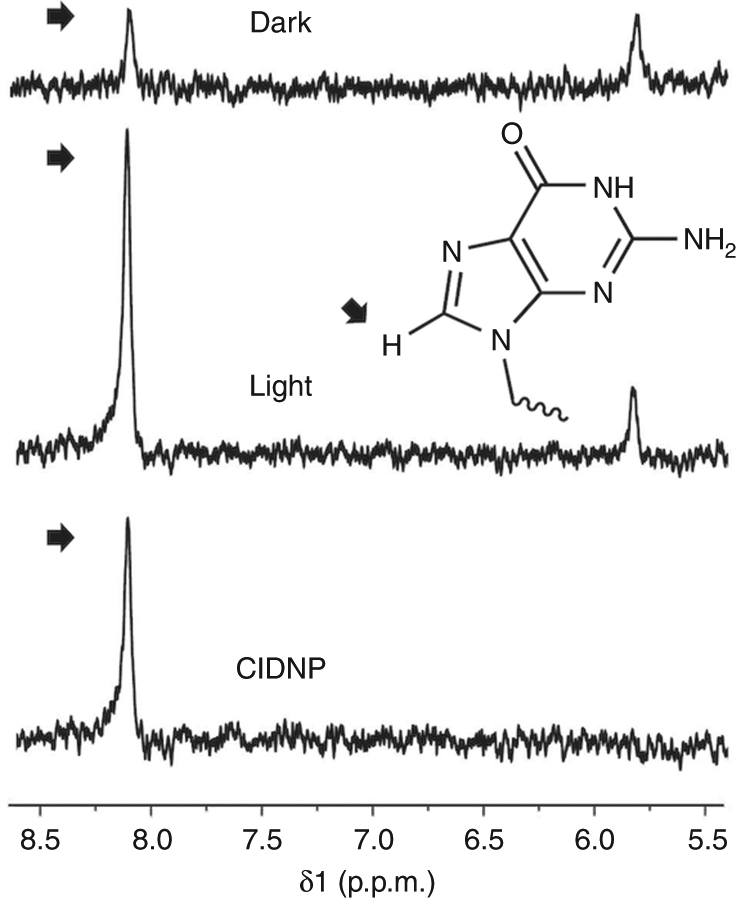
Photo-CIDNP effects on nucleotides. The H8 proton (black arrow) of guanosine mononucleotide (GMP) experiences a strong absorptive signal enhancement upon irradiation in the presence of flavin mononucleotide (FMN). Top: dark spectra. Middle: irradiated spectra. Bottom: CIDNP spectra, shown as the difference (light minus dark). The concentrations were 20 mM GMP and 0.5 mM FMN

We carried out photo-CIDNP investigations on the guanosine monophosphate nucleotide (GMP), since the H8 of the ring is reported to experience a signal enhancement upon light irradiation in the presence of sub-milimolar amounts of flavin mononucleotide (FMN)^40^. Accordingly, 20 mM (7.2 μg, 20 nmol) of GMP were irradiated at 180 mW during 1 s in the presence of 0.5 mM (0.9 μg, 0.5 nmol) of FMN. This resulted in the photo-CIDNP spectra shown in Fig. 2, which is a clear proof of concept of our setup to carry out photo-CIDNP experiments using microcoils. By measuring the signal-to-noise (SNR) ratio between the H8 signal in the light and dark spectra, a ~7-fold ^1^H photo-CIDNP enhancement is observed for GMP under these conditions.

### Photo-CIDNP microcoils under high optical densities

To date,  flavin concentrations in photo-CIDNP experiments were restricted to the sub-mM range (ca. 0.2 mM) due to their high optical densities in solution at higher concentrations^21, 27, 33, 34, 41, 42^. We argue here that increasing the concentration of flavins to the mM range, close to its aggregation limit (ca. 2 mM), would allow efficient photo-CIDNP by virtue of the small volumes afforded by microcoils alleviating the concentration restrictions.

**Fig. 3:**
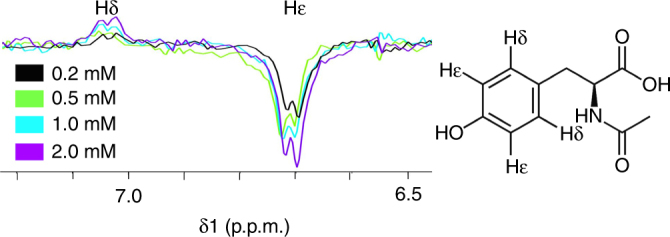
Uniform illumination of high optical density samples. The intensity of the Hε protons of 5 mM N-Acetyl-L-Tyrosine (AcTyr) are shown for increasing concentrations of photosensitizer (FMN). Above 0.5 mM FMN the resulting solutions are of high optical densities, but the small active volume (1 μL) allows a uniform light penetration. Increasing FMN concentrations (from 0.2 to 2.0 mM, see legend) results in higher hyperpolarization effects on the Hε protons of AcTyr

To explore this hypothesis, we performed a series of photo-CIDNP experiments to study the intensity changes on the Hε signal of N-acetyl-L-tyrosine (AcTyr) in solutions containing different FMN concentrations of increasing optical densities; namely, 0.2, 0.5, 1.0, and 2.0 mM, while keeping constant the concentration of AcTyr to 5 mM. Analogous to our experiments on GMP described above, seven microlitres of each sample were injected into the microfluidic channel and irradiated at 180 mW during 6 s. The duration of the laser pulse was chosen according to literature precedents, where a light pulse of 6.4 s of duration yielded the maximum signal enhancement on photo-CIDNP experiments using 3-fluorotyrosine^25^. This assumption was confirmed for our particular setup by optimizing the duration of the light pulse for our experimental conditions (Supplementary Figure 1). The resulting spectra confirmed our hypothesis that, despite of a prominent increase in optical densities when moving from 0.2 to 2.0 mM in flavin concentration, the signal enhancement obtained increases with an increasing concentration of photosensitizer molecules (Fig. 3). This proves that the small active volume located in a microfluidic channel of high surface-to-volume ratio is efficiently, uniformly irradiated using low-power sources regardless of the optical density of the sample. In fact, when moving from a normal NMR tube (*Ø* = 5 mm) to a microchannel (*Ø* = 650 μm), the Lambert-Beer law predicts an order of magnitude decrease in absorption at equimolar concentration, and hence the opportunity to increase the photosensitizer concentration accordingly. The (SNR) ratio corresponding to the Hδ signal of AcTyr as measured from the dark and light spectra represents a ~6-fold ^1^H CIDNP enhancement under high optical density conditions.

### Continuous flow photo-CIDNP experiments on microcoils

Having shown that the small active volumes afforded by microcoils allow precise, uniform light penetration to carry out photo-CIDNP experiments with low-power light sources, we next pursued overcoming the second and major challenge in photo-CIDNP; namely, to avoid the accumulation of photo-degraded flavin in the detection region in a simple yet efficient way. To this end, we exploited the versatility of customized microcoils to work under continuous flow conditions. This setup has significant advantages with respect to previous attempts reported in the literature to prevent flavin degradation^29, 35, 43^, since one can get rid of it by simply flushing continuous fresh sample through the active volume during irradiation.

**Fig. 4:**
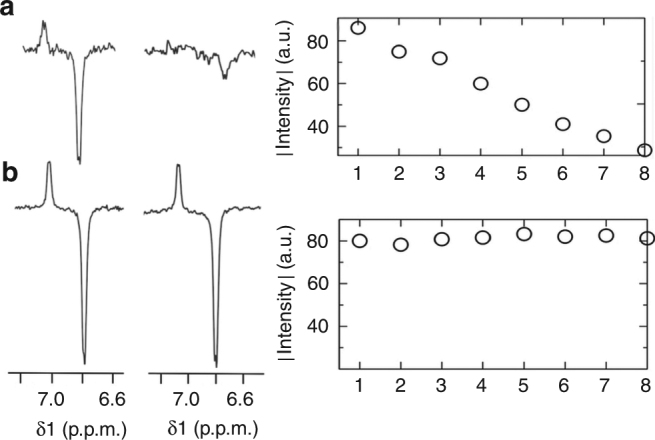
Photo-CIDNP of AcTyr under continuous illumination. **a** Array of eight consecutive (not on-flow)  experiments (28 scans each). Continuous irradiation of the active volume during the whole experiment causes flavin photodegradation. Left of panel: spectra corresponding to the first (1st) and last (8th) experiments showing the impact of degraded flavin on the AcTyr CIDNP signal. Right of panel: Plot of the AcTyr CIDNP signal intensity of the eight consecutive experiments. The increasing signal decay occurs due to gradual accumulation of degraded flavin within the detection volume. **b** Array of eight consecutive on-flow experiments (28 scans each) under continuous illumination. Left of panel: spectra corresponding to the first (1st) and last (8th) experiments showing the benefits of pumping fresh sample on the AcTyr CIDNP signal. Right of panel: Plot of the CIDNP AcTyr signal intensity at the end of each of the eight consecutive experiments. The AcTyr CIDNP signal does not decrease over the course of the experiment since degraded flavin is continuously replaced by fresh sample. Both experiments were performed with the same acquisition parameters and experimental conditions using 5 mM AcTyr and 1 mM FMN in 100% D_2_O

To prove this concept, we recorded a series of photo-CIDNP spectra on 5 mM AcTyr in the presence of 1.0 mM FMN. Under static conditions, continuous irradiation during 12 min causes almost complete flavin degradation. This time of irradiation corresponds to acquisition of 8 consecutive spectra with 28 scans or accumulations each. Due to flavin degradation, a drastic decrease in the intensity of the Hε CIDNP signal of AcTyr is observed between the first and last spectra under these circumstances (Fig. 4a, left panel). Plotting the intensity of the CIDNP signal from each of the eight spectra shows its gradual decay over the course of the experiment (Fig. 4a, right panel). In a second experiment identically performed, but this time pumping fresh sample at a flow rate of 5 μL/min, the signal enhancement remained fairly constant for each of the eight consecutive photo-CIDNP spectra, as can be seen in Fig. 4b, where the first and last spectra show an equal polarization for the Hε protons of AcTyr. Accordingly, no intensity decay is observed when plotting the intensities of the Hε signals in the consecutive experiments (Fig. 4b, right panel), which confirms our hypothesis that working under continuous flow conditions fully prevents accumulation of degraded flavin within the detection region, even when the light source remains switched on during the whole experiment.

The observation that the SNR of the AcTyr CIDNP signal is better under continuous flow conditions (Fig. 4b) with respect to the static experiments (Fig. 4a) is the result of two distinct effects enabled by flow conditions. On the one hand, it is reported that flow intrinsically increases the SNR with respect to static conditions as it ensures pre-equilibration of all sample nuclei and decreases the effective relaxation time^44^. On the other hand, each of the experiments shown in Fig. 4 consists of 28 scans, a time during which degraded flavin is already accumulating within the active volume that is subjected to continuous irradiation. Therefore, under static conditions signal averaging within each experiment comprises increasing amounts of degraded flavin that does not contribute to the CIDNP signal, which is not the case under continuous flow conditions where the sample located within the active volume is continuously refreshed. However, one should bear in mind that static conditions use just 1 μL of material, a much lower amount than that required for flow conditions. Nevertheless, only 60 μL of sample (67 μg of AcTyr) were required to record this large number (>200) of 1D spectra under flow conditions. Considering that conventional photo-CIDNP experiments employ light pulses alternated with 'dark' periods of several seconds (usually >15 s, see Supplementary Figure 2 for pulsed irradiation on microcoils), the use of continuous irradiation under continuous flow conditions results in much shorter experiment times with much lower amounts of sample.

### Efficient mixing by on-flow photo-CIDNP on microcoils

A key feature of photo-CIDNP experiments is the unique capability to non-invasively monitor solvent accessibility of aromatic residues in peptides and proteins^21, 27, 45–50^, whose signal intensities or chemical shifts are modified according to changes in their solvent exposure or burial. Accordingly, we tested our system’s ability to detect such changes by monitoring the Hε resonance of the Tyr moiety in two different scenarios; namely, (i) when the amino acid becomes less exposed to the solvent using an encapsulating agent, and (ii) when it becomes more exposed to the solvent using a denaturizing agent in the context of a well-folded peptide. For the first experiment we studied the encapsulation of AcTyr by β-cyclodextrin (β-CyD). In the second experiment we followed changes of two tyrosine residues in the 197-TGWKKIADKWYYFN-210 sequence, which corresponds to the pneumococcal autolysin protein LytA (LytA_239–252_), in the presence and absence of 4.5 M urea.

In order to carry out these studies under continuous flow conditions that would benefit from all the advantages exposed in the previous section, we further optimized the NMR chip device with a new design that contains two inlets (two entries for sample, see Fig. 1). The idea beneath this design consists of enabling rapid and efficient mixing of the two samples prior to irradiation and detection, so that conventional elaborated setups^37, 51^ can be avoided. Supplementary Figure 3 proves the quality of the mixing by fluorescence microscopy. The system setup consists of two syringes, a first one containing 2 mM FMN and the target sample (AcTyr or LytA_239–252_), and a second one with β-CyD or urea, respectively, to promote solvent burial or exposure of the corresponding target molecule. A flow rate of 2.5 μL/min was used to load each sample through the corresponding inlet, so as to have a net entry of 5 μL/min, according to the flow conditions used in the previous section.

**Fig. 5:**
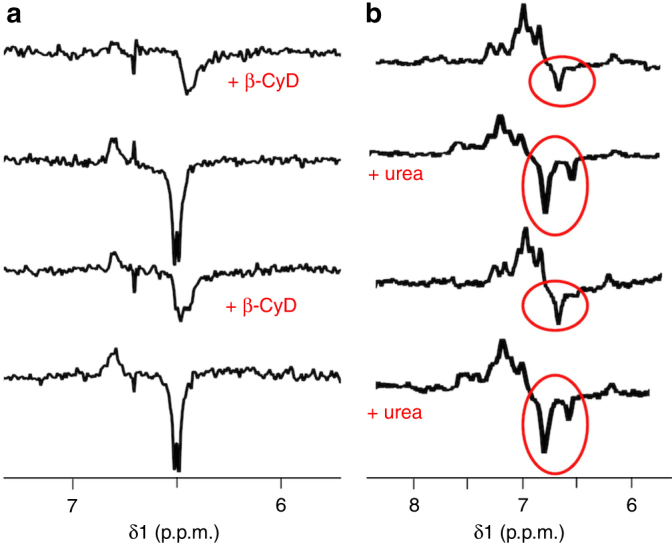
Efficient mixing on microcoils for sophisticated photo-CIDNP applications. **a** An experiment to promote solvent burial of a polarizable molecule. Mixing of 10 mM AcTyr and 2 mM FMN (through a syringe, ‘1’) with 30 mM β-cyclodextrin (β-CyD) (using another syringe, ‘2’), results in a mixture of 5 mM AcTyr, 1 mM FMN and 15 mM β-CyD in the detection region in which the amino acid becomes encapsulated. Its lower solvent exposure (and therefore accessibility to FMN), causes the AcTyr signal to broaden and shift (see also Supplementary Figure 3). **b** An experiment to promote solvent exposure of a polarizable molecule. Mixing of 4 mM LytA_239–252_ and 2 mM FMN (syringe 1) with 9 M urea (syringe 2), results in a mixture of 2 mM LytA_239–252_, 1 mM FMN and 4.5 M urea in the detection region in which LytA_239–252_ loses the native structure and the two tyrosines become solvent exposed. The higher accessibility to FMN yields two signals corresponding to the Hε protons of two distinct tyrosines show up in the spectra. Note the highly reproducible pattern in both cases when the mixing is turned on and off

Figure 5a shows the photo-CIDNP spectra corresponding to the outcome of the first experiment (mixing of AcTyr and β-CyD to promote solvent burial). When the syringe containing β-CyD is switched on, both β-CyD and AcTyr are in contact, which results in the encapsulation of the amino acid. Accordingly, the emissive signals of its Hε protons slightly shift, broaden, and lose intensity as a consequence of the supramolecular interaction that lowers the solvent accessibility of AcTyr (see also Supplementary Figure 4). On the other hand, when the syringe pump containing β-CyD is switched off, the resulting photo-CIDNP spectra is identical to that shown in Fig. 4 for freely, solvent-exposed AcTyr. This behaviour is consistently reproduced.

In the second experiment (mixing of LytA_239–252_ and urea to promote solvent exposure), switching on the syringe containing urea allows mixing of urea and LytA_239–252_. Therefore, the high concentration of denaturizing agent causes a loss of native structure that results in unfolded conformations in which the two tyrosines are solvent exposed. Consequently, two peaks corresponding to the Hε protons of two tyrosines appear in the photo-CIDNP spectra under these circumstances (Fig. 5b). Although there is no deposited structure for this system in the Protein Data Bank, the original paper reporting the NMR solution structure of LytA_239–252_ clearly illustrates the two tyrosines as having two radically different atomic environments^52^: Tyr249 (Y249) is clustered against side chains from F251, I244, and K247, establishing a number of intermolecular contacts. In contrast, Tyr250 (Y250) is highly exposed to the solvent. These reported data reconcile our observation of one and two tyrosine signals when urea is absent and present, respectively.

### Microcoils and photo-CIDNP enable sub-picomole detection

**Fig. 6:**
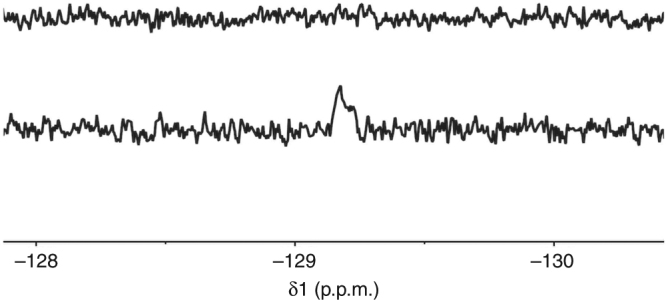
Sub-picomole detection combining photo-CIDNP and microcoil NMR technology. Top: ^19^F NMR spectrum of a sample containing 0.4 mM of p-fluorophenol, which cannot be detected. Bottom of figure: The photo-CIDNP spectrum under flow conditions of a 0.8 μM p-fluorophenol sample. This corresponds to detection of just 0.8 picomole of material per μL. The number of accumulated scans is 14 in both cases

To explore the limit-of-detection that can be achieved by combining the enhanced mass sensitivity of microcoils with photo-CIDNP, we bear in mind that CIDNP intensities are directly related to the magnitude of the hyperfine couplings in the corresponding transient radicals^24, 25^. In this regard, ^19^F photo-CIDNP NMR studies of fluorinated compounds are very convenient for two reasons; namely, the natural abundance of ^19^F (100%), and the unusually large hyperfine coupling constant of this nuclide in p-fluorophenol^53–55^. This prompted us to prepare solutions of different concentrations and make dilutions until we could not detect any ^19^F CIDNP signal upon irradiation. We reached this limit at a sample concentration of 0.8 μM, which considering the 1 μL active volume corresponds to sub-picomole amount of material. Figure 6 (top) shows the dark spectrum of a sample containing 0.4 mM of p-fluorophenol (0.4 nmol), which cannot be detected as it falls out the LOD of the microcoil (2 nmol in absence of CIDNP, see Methods section). The situation is drastically different when the sample is irradiated in the presence of equimolar amounts of flavin, to such extent that even a sample containing 0.8 μM of p-fluorophenol could be detected. Figure 6 (bottom) shows the corresponding ^19^F photo-CIDNP spectrum, where it is possible to detect such a diluted sample that corresponds to just 0.8 pmol of material per μL.

## Discussion

An application of microcoil technology that boosts NMR mass- and concentration sensitivity by combining these devices with a hyperpolarization scheme, photo-CIDNP, is presented. Performing these experiments on reduced-diameter NMR coils smartly overcomes traditional challenges of the technique; namely, high-power irradiation, lack of uniform irradiation, and accumulation of degraded flavin in the detection region. The combination of a PDMS matrix with low-power light sources employed in our setup alleviates heating effects, especially in light of the low heat-conductivity properties of PDMS. It is remarkable that the use of LED-based illumination was recently devised to irradiate samples at lower power for conventional 5-mm NMR tubes, as discussed in the introduction. In our setup, we have shown that the small active volumes and the possibility to work under continuous flow conditions prevent these challenges, all this within the framework of a very simple setup in which the microcoils employed are easily manufactured within only one day^19^, and just using basic lab equipment and materials. Furthermore, the combination of continuous flow conditions and low-power irradiation allows working with continuous irradiation, which removes the need to trigger light pulses from the spectrometer, reducing experiment time, and to resort to complex pulse sequences.

In summary, we present the development of a simple, powerful and low-cost setup to perform photo-CIDNP with high efficacy on 1 µL detection volumes and with uniform illumination of the entire sample and avoiding high-power laser that overcomes current limitations in the field. This makes photo-CIDNP more accessible to the scientific community and may open new opportunities based on a rapid, efficient mixing of samples to develop novel applications. Current possibilities under investigation by virtue of the possibilities of the setup presented here, include expanding LODs to the sub-picomole level, along with the possibility of performing 2D heteronuclear experiments owing to our recently developed broadband, non-resonant microcoils with active volumes in the nanolitre range^56^.

## Methods

### Characterization of the transceiver solenoidal NMR microcoil

The solenoidal coil manufactured to perform the experiments presented in this work consists of a coated copper wire (100 μm diameter) encompassing a cylindrical volume with *Ø* = 650 μm. The resulting coil has a total length of 3 mm, and covers a detection volume of 1 μL. The microcoil is mounted on the probe and electrically connected to three capacitors that allow tuning and matching to ^1^H and ^19^F frequencies, similar to the setup described by Popovic and coworkers^12^, as illustrated in Fig. 1 (see magnified region). A nutation curve is shown in Supplementary Figure 5 to highlight the excellent B_1_ field homogeneity of the coil.

To characterize the performance of this transceiver microcoil, we have used the mass and concentration sensitivity (*S*_*m*_ and *S*_*c*_, respectively) and the normalized (mass and concentration) limit-of-detection (*n*LOD_*m*_ and *n*LOD_*m*_) parameters, which are appropriate indicators for mass-limited samples and to compare microcoil probes of different active volumes and whose definitions can be found in ref. ^8^. Using a sample consisting of 20 mM GMP in 100% D_2_O, the following values were obtained from a single-scan experiment with an acquisition time of 0.5 s: *S*_*m*_ = 1433 (2790) SNR μmol^−1^ s^−1/2^, *S*_*c*_ = 1.433 (0.014) SNR mM^−1^ s^−1/2^, *n*LOD_*m*_ = 2 (55) nmol s^−1/2^ SNR^−1^, and *n*LOD_*c*_ = 2 (0.27) mM s^1/2^ SNR^−1^. The values in parenthesis correspond to the 5-nL solenoidal microprobe reported by Olson et al.^4^, and have been already scaled up to 400 MHz for a proper comparison using the B_0_^7/4^ factor^8, 56^. As expected, the smaller the active volume, the higher the mass sensitivity, and the lower the concentration sensitivity.

### Photo-CIDNP experiments

^1^H photo-CIDNP experiments on microcoils were carried out in a 9.4T narrow-bore Oxford instruments magnet (400 MHz ^1^H Larmor frequency and 376 MHz ^19^F Larmor frequency) INOVA NMR spectrometer (Oxford, Agilent). A sacrificed commercial probe was used as the scaffold onto which the hand-made solenoids embedded in the PDMS matrix were mounted. The procedure to customize these microcoils is described in detail elsewhere^19^. The low-power light source used for the experiments consists of a laser diode emitting at 450 nm, operating at 1.6 W output power. The optical power is lowered to the milliWatt range by using a potentiometer that regulates the current through the diode, and a photometer was used to monitor the desired power from the light beam coming out of a 1 mm (internal diameter) optical fibre. This current controller (Thorlabs, LDC 205C/240C) can be switched on and off to regulate the duration and intensity of the light pulse manually (continuous irradiation) or by a TTL signal directly from the spectrometer, therefore to be controlled by the user through the pulse sequence, for light pulses photo-CIDNP experiments. A temperature controller (Thorlabs, TED 200C) is also used to ensure no variation of the output light power.

For experiments performed under flow conditions, a total flow rate of 5 μL/min is created using syringe pumps as depicted in Fig. 1. Since the longer experiment shown in Fig. 4 takes 12 min to be completed, no more than 60 μL of total sample is required to carry out these measurements. In practice, typically a 100 μL glass syringe is used, and the flow is handled using syringe pumps.

All the samples (GMP, FMN, AcTyr, and β**-**CyD) were purchased from Sigma Aldrich and directly dissolved in 100% D_2_O to the concentrations specified for each experiment. The peptide corresponding to LytA_190-213_ was obtained from R-Peptide, whose identity and purity (>95%) were confirmed by HPLC and mass spectrometry. Spectra were processed in VnmrJ, and all signal-to-noise ratios (SNRs) were determined as the maximum height of the peak divided by the root-mean-square noise of the baseline using the SNR peak calculator script implemented in MestReNova (http://www.mestrec.com).

### Data availability

All relevant data supporting the results of this study are available from the corresponding author upon request.

## Electronic supplementary material

Supplementary Information

## References

[1] Wüthrich K (2003). NMR studies of structure and function of biological macromolecules (nobel lecture). Angew. Chem. Int. Ed..

[2] Kovacs H, Moskau D, Spraul M (2005). Cryogenically cooled probes—a leap in NMR technology. Prog. Nucl. Magn. Reson. Spectrosc..

[3] Ardenkjaer-Larsen JH (2003). Increase in signal-to-noise ratio of >10,000 times in liquid-state NMR. Proc. Natl Acad. Sci. USA.

[4] Olson DL, Peck TL, Webb AG, Magin RL, Sweedler JV (1995). High-resolution microcoil ^1^H-NMR for mass-limited, nanoliter-volume samples. Science.

[5] Webb AG (2013). Radiofrequency microcoils for magnetic resonance imaging and spectroscopy. J. Magn. Reson..

[6] Fratila RM, Velders AH (2011). Small-volume nuclear magnetic resonance spectroscopy. Annu. Rev. Anal. Chem..

[7] Jones CJ, Larive CK (2012). Could smaller really be better? Current and future trends in high-resolution microcoil NMR spectroscopy. Anal. Bioanal. Chem..

[8] Lacey ME, Subramanian R, Olson DL, Webb AG, Sweedler JV (1999). High-resolution NMR spectroscopy of sample volumes from 1 nL to 10 &mgr;L. Chem. Rev..

[9] Kentgens AP (2008). High-resolution liquid- and solid-state nuclear magnetic resonance of nanoliter sample volumes using microcoil detectors. J. Chem. Phys..

[10] Boero G (2003). Electron-spin resonance probe based on a 100 μm planar microcoil. Rev. Sci. Instrum..

[11] Ehrmann K (2006). Sample patterning on NMR surface microcoils. J. Magn. Reson..

[12] Massin C (2003). Planar microcoil-based microfluidic NMR probes. J. Magn. Reson..

[13] Ehrmann K (2007). NMR spectroscopy and perfusion of mammalian cells using surface microprobes. Lab. Chip..

[14] Gomez MV, Reinhoudt DN, Velders AH (2008). Supramolecular interactions at the picomole level studied by 19F NMR spectroscopy in a microfluidic chip. Small.

[15] Gomez MV (2010). On-line monitoring of a microwave-assisted chemical reaction by nanolitre NMR-spectroscopy. Chem. Commun..

[16] Gomez MV (2015). Determination of kinetic parameters within a single nonisothermal on-flow experiment by nanoliter NMR spectroscopy. Anal. Chem..

[17] Causier A, Carret G, Boutin C, Berthelot T, Berthault P (2015). 3D-printed system optimizing dissolution of hyperpolarized gaseous species for micro-sized NMR. Lab. Chip..

[18] Finch G, Yilmaz A, Utz M (2016). An optimised detector for in-situ high-resolution NMR in microfluidic devices. J. Magn. Reson..

[19] Saggiomo V, Velders AH (2015). Simple 3D printed scaffold-removal method for the fabrication of intricate microfluidic devices. Adv. Sci..

[20] Hore J, Broadhurst RW (1993). Photo-CIDNP of biopolymers. Progress. Nucl. Magn. Reson. Spectrosc..

[21] Kaptein R, Dijkstra K, Nicolay K (1978). Laser photo-CIDNP as a surface probe for proteins in solution. Nature.

[22] Abragam A, Goldman M (1978). Principles of dynamic nuclear polarisation. Rep. Progress. Phys..

[23] Goez, M. in: *Advances in Photochemistry 63-163* (John Wiley & Sons, Inc., Hoboken, NJ, USA. 2007).

[24] Hore PJ, Egmond MR, Edzes HT, Kaptein R (1982). Cross-relaxation effects in the photo-CIDNP spectra of amino acids and proteins. J. Magn. Reson. (1969).

[25] Kuprov I, Hore PJ (2004). Chemically amplified 19F-1H nuclear Overhauser effects. J. Magn. Reson..

[26] Mok KH (2007). A pre-existing hydrophobic collapse in the unfolded state of an ultrafast folding protein. Nature.

[27] Mok KH (2003). Rapid sample-mixing technique for transient NMR and photo-CIDNP spectroscopy: applications to real-time protein folding. J. Am. Chem. Soc..

[28] Kuprov I, Hore PJ (2004). Uniform illumination of optically dense NMR samples. J. Magn. Reson..

[29] Maeda K (2000). Improved photo-CIDNP methods for studying protein structure and folding. J. Biomol. Nmr..

[30] Kocha T, Yamaguchi M, Ohtaki H, Fukuda T, Aoyagi T (1997). Hydrogen peroxide-mediated degradation of protein: different oxidation modes of copper- and iron-dependent hydroxyl radicals on the degradation of albumin. Biochim. Biophys. Acta.

[31] Zmijewski JW (2010). Exposure to hydrogen peroxide induces oxidation and activation of AMP-activated protein kinase. J. Biol. Chem..

[32] Goez M, Kuprov I, Hun Mok K, Hore PJ (2006). Novel pulse sequences for time-resolved photo-CIDNP. Mol. Phys..

[33] Lee JH, Sekhar A, Cavagnero S (2011). 1H-Detected 13C photo-CIDNP as a sensitivity enhancement tool in solution NMR. J. Am. Chem. Soc..

[34] Sekhar A, Cavagnero S (2009). EPIC- and CHANCE-HSQC: two 15N-photo-CIDNP-enhanced pulse sequences for the sensitive detection of solvent-exposed tryptophan. J. Magn. Reson..

[35] Lee JH, Cavagnero S (2013). A novel tri-enzyme system in combination with laser-driven NMR enables efficient nuclear polarization of biomolecules in solution. J. Phys. Chem. B.

[36] Dolinski ND (2017). A Versatile Approach for In Situ Monitoring of Photoswitches and Photopolymerizations. ChemPhotoChem.

[37] Feldmeier C, Bartling H, Riedle E, Gschwind RM (2013). LED based NMR illumination device for mechanistic studies on photochemical reactions--versatile and simple, yet surprisingly powerful. J. Magn. Reson..

[38] Stadler, E., Eibel, A., Neshchadin, D. & Gescheidt, G. in *Zeitschrift für Physikalische Chemie* **231**, 625–636 (2017).

[39] Li, M. & Kim, D. P. Silicate glass coated microchannels through a phase conversion process for glass-like electrokinetic performance. *Lab Chip* **11**, 1126–1131 (2011). 10.1039/c0lc00522c 21301730

[40] Stob S, Scheek RM, Kaptein R (1989). Photo-CIDNP in guanine nucleic acid derivatives. a mechanistic study. Photochem. Photobiol..

[41] Hore PJ, Volbeda A, Dijkstra K, Kaptein R (1982). Photoreduction of flavin by NADH. A flash photolysis photo-CIDNP study. J. Am. Chem. Soc..

[42] Nisimoto Y, Hayashi F, Akutsu H, Kyogoku Y, Shibata Y (1984). Photochemically induced dynamic nuclear polarization study on microsomal NADPH-cytochrome P-450 reductase. J. Biol. Chem..

[43] Connolly PJ, Hoch JC (1991). Photochemical degradation of tryptophan residues during CIDNP experiments. J. Magn. Reson. (1969).

[44] Laude DA, Wilkins CL (1986). Applications of a recycled-flow Fourier transform nuclear magnetic resonance system: molecular weight determination of siloxane polymers by silicon-29 NMR. Macromolecules.

[45] Muszkat KA, Gilon C (1978). CIDNP in tyrosyl protons of luliberin. Nature.

[46] Balbach J (1995). Following protein folding in real time using NMR spectroscopy. Nat. Struct. Biol..

[47] Rubinstenn G (1998). Structural and dynamic changes of photoactive yellow protein during its photocycle in solution. Nat. Struct. Biol..

[48] Wirmer J, Kühn T, Schwalbe H (2001). Millisecond time resolved Photo-CIDNP NMR reveals a non-native folding intermediate on the ion-induced refolding pathway of bovine α-lactalbumin. Angew. Chem. Int. Ed..

[49] Lyon CE, Suh ES, Dobson CM, Hore PJ (2002). Probing the exposure of tyrosine and tryptophan residues in partially folded proteins and folding intermediates by CIDNP pulse-labeling. J. Am. Chem. Soc..

[50] Khan F, Kuprov I, Craggs TD, Hore PJ, Jackson SE (2006). 19F NMR studies of the native and denatured states of green fluorescent protein. J. Am. Chem. Soc..

[51] Hore PJ, Winder SL, Roberts CH, Dobson CM (1997). Stopped-flow photo-CIDNP observation of protein folding. J. Am. Chem. Soc..

[52] Zamora-Carreras H (2015). Micelle-triggered beta-hairpin to alpha-helix transition in a 14-residue peptide from a choline-binding repeat of the pneumococcal autolysin LytA. Chemistry.

[53] Kuprov I, Craggs TD, Jackson SE, Hore PJ (2007). Spin relaxation effects in photochemically induced dynamic nuclear polarization spectroscopy of nuclei with strongly anisotropic hyperfine couplings. J. Am. Chem. Soc..

[54] Perrier S, Mugeniwabagara E, Kirsch-De Mesmaeker A, Hore PJ, Luhmer M (2009). Exploring photoreactions between polyazaaromatic Ru(II) complexes and biomolecules by chemically induced dynamic nuclear polarization measurements. J. Am. Chem. Soc..

[55] Tomkiewicz M, McAlpine RD, Cocivera M (1972). Photooxidation and Decarboxylation of Tyrosine Studied by E.P.R. and C.I.D.N.P. Techniques. Can. J. Chem..

[56] Fratila RM, Gomez MV, Sykora S, Velders AH (2014). Multinuclear nanoliter one-dimensional and two-dimensional NMR spectroscopy with a single non-resonant microcoil. Nat. Commun..

