# Supplementary material for: Pushing nuclear magnetic resonance sensitivity limits with microfluidics and photo-chemically induced dynamic nuclear polarization
Source: Nat Commun. 2018 Jan 9;9:108. doi: 10.1038/s41467-017-02575-0 (PMC5760532; doi:10.1038/s41467-017-02575-0)
Supplement: Supplementary file 1 — Supplementary Information [file 41467_2017_2575_MOESM1_ESM.pdf]

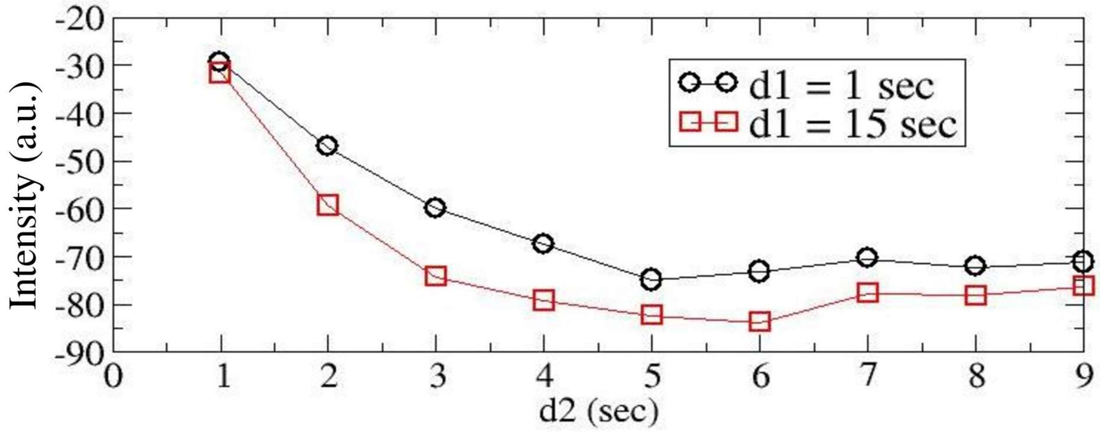

Supplementary Figure 1. Optimizing the duration of the light pulse.

The optimum value for the irradiation time (d2) was determined for two different recycling delays (d1) of 1 and 15 seconds, after 8 scans and an acquisition time of 0.5 seconds per scan. The latter value is preferred, since it allows re-equilibration of the spin populations between light flashes. The sample consisted of 5 mM AcTyr and 1 mM FMN, and the CIDNP signal intensity of the Hε protons obtained for each individual experiment is plotted. These peaks are in emission (see **Figure 3–5** in the main text) and therefore, negative intensities are plotted.

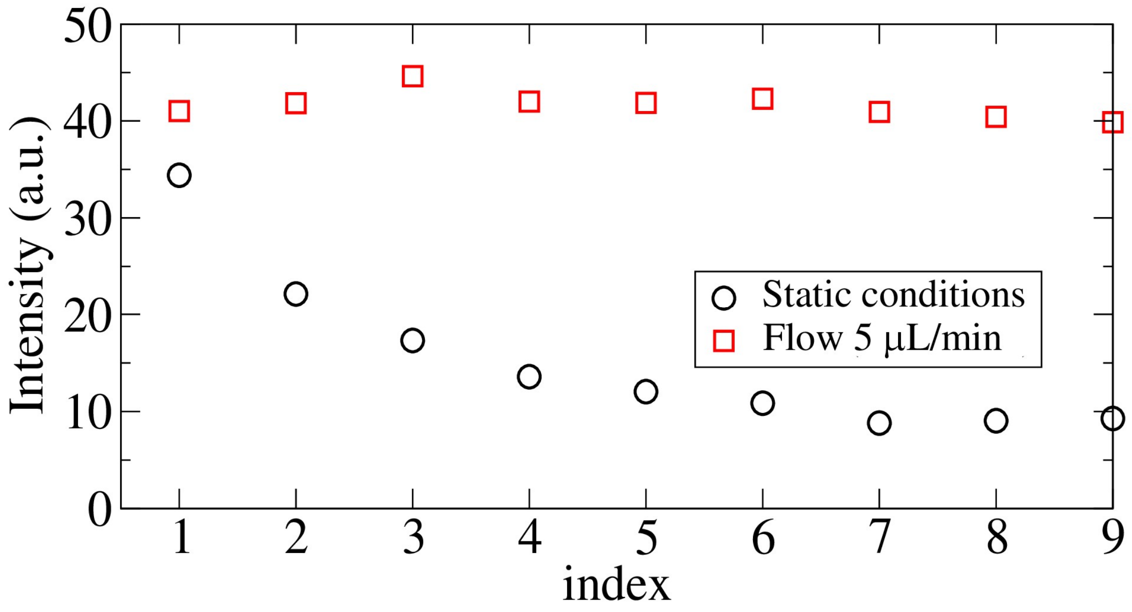

Supplementary Figure 2. FMN degradation under finite light pulses.

The intensity of the Hε protons of 5 mM AcTyr in the presence of 1 mM FMN is shown when using light pulses of finite duration (6 seconds). Each experiment consists of 4 scans. Light pulses alternate with "dark" periods of 15 seconds (i.e., repeatedly 6 seconds of light – 15 seconds of dark – 6 seconds of light – 15 seconds of dark). In the case of the experiments under continuous flow conditions (red squares), which were performed using the same acquisition parameters, no flavin degradation occurs due to a fresh, continuous load of fresh sample that removes degraded products from the detection region of the coil. Note that the difference in SNR between static and flow experiments is not as pronounced as in **Figure 4** (main text) for the first data point since each experiment here consist of 4 scans, and not 28, and therefore there is less flavin degradation. Accordingly, the second data point already illustrates a larger difference.

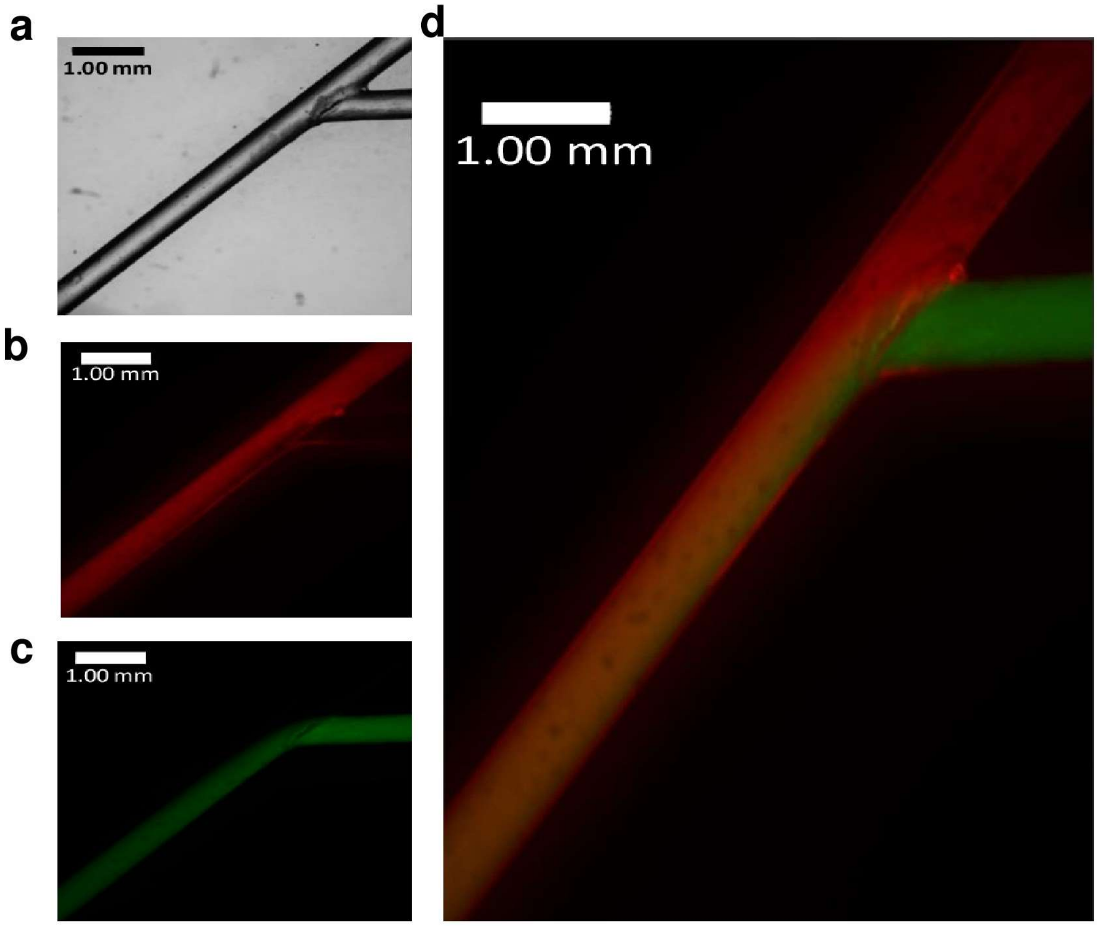

Supplementary Figure 3. Fluorescent evidence of efficient mixing.

(a) Fluorescence microscopy images of the microchannel. Two fluorescent probes, **(b)** rhodamine (red) and **(c)** fluorescein (green) were injected to confirm the quality of the mixing **(d)**. The bar scale in all figures is 1.00 mm. These images were obtained using a Leica DMi8 microscope with 2.5X. A RHOD filter was used for visualization of rhodamine, and a FITC one was employed in the case of fluorescein. Two different exposure times were employed; namely, 100 ms for rhodamine, and 75 ms for fluorescein.

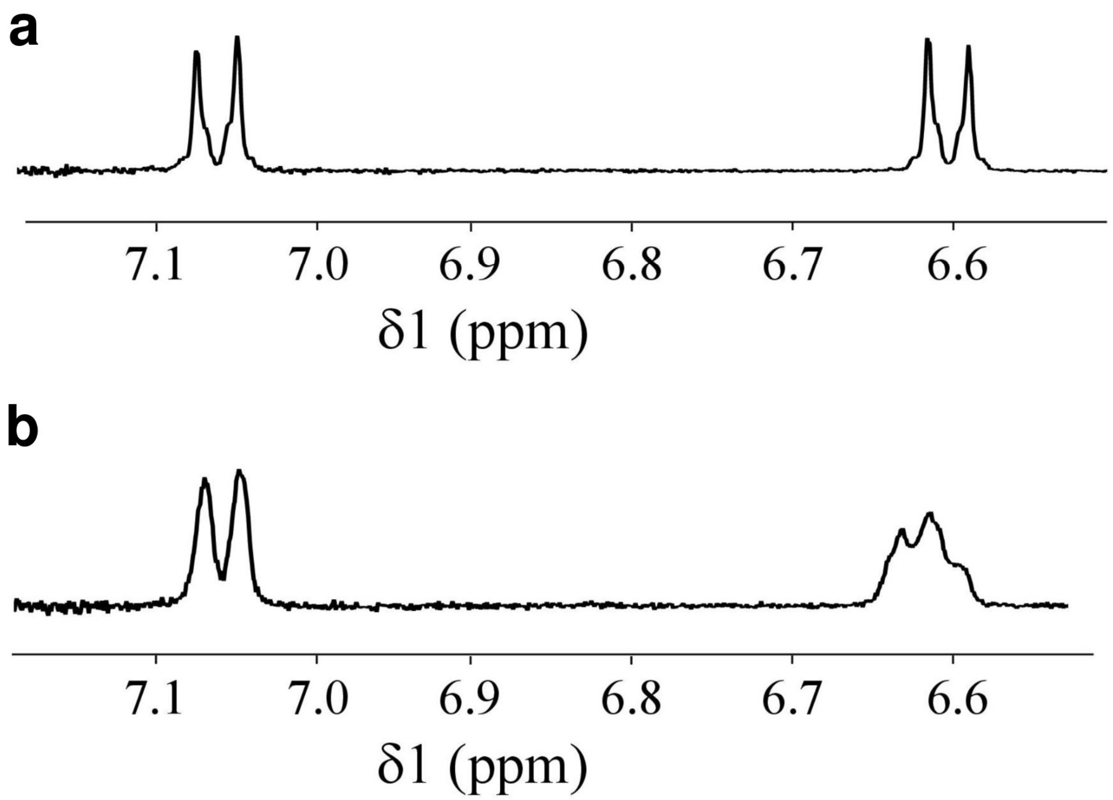

Supplementary Figure 4. Macromolecular interaction between AcTyr and β-CyD.

(**a**) A sample containing 10 mM AcTyr in 100% D_2O.

(**b**) Addition of 30 mM β-CyD causes a strong perturbation of the Hε protons of AcTyr.

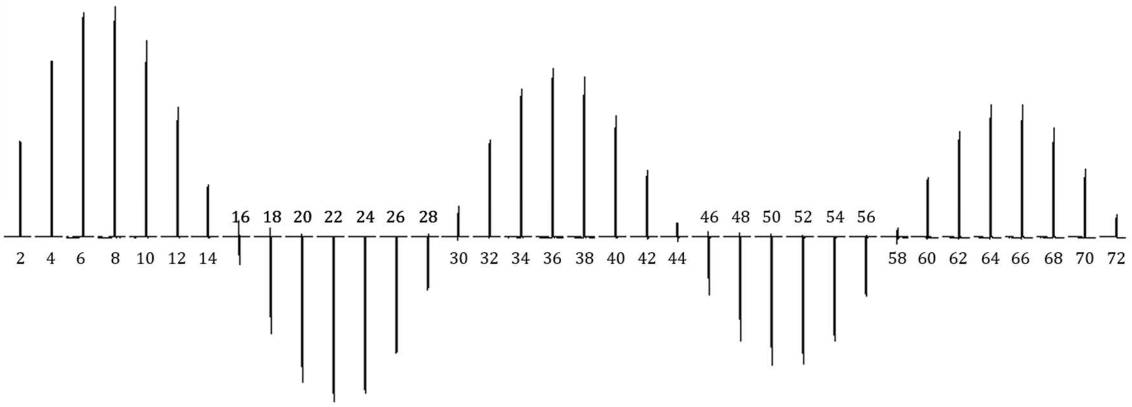

Supplementary Figure 5. Nutation curve to illustrate field homogeneity.

An array of the pulse length is shown as obtained from the following parameters: acquisition time of 0.5 seconds, recycling delay (d1) of 3 seconds. The ^{1}H 90º pulse was determined to be of 7.5 μs. Sample was H_2O.
